# Editorial for the Special Issue on Flash Memory Devices

**DOI:** 10.3390/mi12121566

**Published:** 2021-12-17

**Authors:** Cristian Zambelli, Rino Micheloni

**Affiliations:** Dipartimento di Ingegneria, Università degli Studi di Ferrara, Via G. Saragat 1, 44122 Ferrara, Italy; rino.micheloni@ieee.org

Flash memory devices represented a breakthrough in the storage industry since their inception in the mid-1980s, and innovation is still ongoing after more than 35 years. They are the largest landscape of storage devices, and we expect more and more advancements in the coming years. The peculiarity of such technology is an inherent flexibility in terms of performance and integration density according to the architecture devised for integration of cells. In the context of code storage applications in the embedded world, automotive microcontrollers, IoT smart devices, and edge AI, we rely on NOR Flash technology. Their density ranges from a few Kbytes up to the Gigabit size. However, when massive data storage is required, NAND Flash memories are a must in a system. NAND Flash can be found in USB and Flash Cards (SD, eMMC), but most of all in Solid-State Drives (SSDs). Since SSDs are extremely demanding in terms of storage capacity, they fueled a new wave of innovation for Flash memories, namely 3D architecture. Today, 3D means that multiple layers (up to almost two hundred, as we speak) of memory cells are manufactured within the same piece of silicon, easily reaching a terabit of storage capacity per chip. This will require a lot of innovation in process technology, materials, circuit design, flash management algorithms, Error Correction Code (ECC), and finally system co-design for new applications such AI and security enforcement.

This Special Issue provides insight on and advancements in Flash memory devices. There are nine papers including one review paper, covering the reliability of 3D NAND Flash devices [1,2,3], the characterization and design of Flash memory cell/string [2,4,5], NOR Flash memories for embedded applications [5], a set of Error Correction Codes and Secondary Correction Algorithms for flash memories [6,7], Flash management through flash signal processing in controllers for Big Data storage [6,7,8], and the impact of Flash memories on Solid State Drives reliability and performance [8,9].

Flash memory devices integrated either with planar or 3D process scheme suffer from major performance and reliability threats that can be handled starting from the first manufacturing process steps. In [1], Spinelli et al. reviewed the phenomenology of random telegraph noise (RTN) in 3D NAND Flash arrays to deeply understand such a time-dependent reliability issue. They pointed out the relevant role played by the polycrystalline nature of the string silicon channels through experimental data and simulation models of the current transport. The RTN features changed significantly in the transition from planar to 3D processes due to the presence of highly defective grain boundaries on percolative current transport in cell channels in combination with the localized nature of the RTN traps. In [2], Ramesh et al. studied the erase operation performance by characterizing the metal gate work function of different metal electrode and high-k dielectric combinations in 3D Flash cell stack integration. They investigated the impact of different thermal treatments on the work function and observed a dipole formation at the metal/high-k and/or high-k/SiO_2_ interfaces. They also concluded that the erase performance of metal/high-k/ONO/Si (MHONOS) capacitors is identical to the gate stack in three-dimensional (3D) NAND Flash, although the work function extraction is convoluted by the dipole formation. In [3], Chen et al. investigated the temperature effects that affect the reliability and performance of NAND flash memories. They characterized Triple-Level Cell (TLC) 3D NAND flash memory chips in a wide temperature range by focusing on the raw bit error rate (RBER) degradation during frequent-write (endurance) and frequent-read (read disturb) working conditions. It was observed that the program time shows strong dependence on the temperature and lifetime degradation induced by cycling and that the RBER can be suppressed at higher temperatures. Read disturb has been found to be more detrimental at low temperatures, but it can be beneficial for RBER recovery at high temperatures.

A successful Flash technology requires a careful design of the cell structure and of the operation modes. In [4], Yi et al. addressed the minimization of the threshold voltage variation of programmed cells by developing a new programming scheme to write the cells from the top array in vertical NAND (VNAND) structures to reach 5 bits per cell storage paradigm. With the aid of Technology-Computer-Aided Design (TCAD), the Z-Interference for this new program algorithm is found to be better than the state of the art by at least 20 mV. Moreover, under scaled cell dimensions, the improvement becomes protruding. In [5], Song et al. incorporated aluminum oxide in tunnel oxide to improve retention characteristics of NOR flash arrays. By adopting the proposed tunneling layers in the NOR flash array, the threshold voltage window after 10 years from programming and erasing (P/E) was improved by 4 V. The validation of the proposed device structure took place by comparing it with another stacked-engineered structure with SiO_2_/Si_3_N_4_/SiO_2_ tunneling layers. Simulations through TCAD were exploited in this context. In addition, to verify that our proposed structure is suitable for NOR flash array, disturbance issues are also carefully investigated.

As Flash architectures scale, their reliability worsens significantly and they require proactive control by using either advanced Error Correction Codes or some secondary correction mechanisms that help the recovery of the corrupted stored information. NAND flash memories are addressed especially in this context. In [6], Zhang et al. proposed a set of machine learning algorithms to accurately predict endurance levels of the memory array, which is of great significance for effectively extending the lifetime of NAND flash memory devices and avoiding serious losses caused by sudden failures. In this work, a multi-class endurance prediction scheme based on the SVM algorithm is proposed, which can predict the remaining endurance level and the RBER at various lifetime points. Feature analysis based on endurance data is used to determine the basic elements of the model and its implementation on a System-on-Chip (SoC) module showing the completion of a single prediction within 37 μs. In [7], He et al. presents a novel neural-network-assisted error correction (ANNAEC) scheme to increase the reliability of multi-level cell (MLC) NAND Flash memory. They propose a relative log-likelihood ratio (LLR) to estimate the actual LLR and transform the bit detection into a clustering problem suitable for a neural network to learn the error characteristics of the NAND flash memory channel. Simulation results show that the proposed scheme can significantly increase the lifetime of NAND flash memories.

The interaction of Flash memories at the system level as currently happens in Solid State Drive (SSD) architectures is also of paramount importance. The physics of devices and the higher abstraction levels of the digital electronics come together in this context. In [8], M. Favalli et al. discussed the data randomization for reducing or suppressing errors. In this work, they proposed a randomization scheme that is easy to implement, cost effective, and fully scalable with memory dimensions and guarantees optimal randomization along the wordline and the bitline dimensions. The method has been validated on commercial off-the-shelf TLC 3D NAND Flash memory. In [9], Du et al. defined garbage collection (GC) as a time-consuming but necessary operation in Flash memories. They performed a comprehensive experimental study in view of a performance cliff that closely relates to Quality of Service (QoS). Through system-level simulations, they found that 3D NAND Flash based SSDs exacerbate the situation by inducing a much higher number of page migrations during GC. To relieve the performance cliff problem, they propose PreGC to assist normal GC. Experimental results show that PreGC can efficiently relieve the performance cliff by reducing the tail latency from the 90th to 99.99th percentiles.

We thank all the authors who submitted their papers to this Special Issue. We would also like to acknowledge all the reviewers, whose careful and timely reviews ensured the quality of this Special Issue.
